# Developing and Validating a Robust RP-HPLC Method for Metoclopramide and Camylofin Simultaneous Analysis Using Response Surface Methodology

**DOI:** 10.1155/ianc/5543392

**Published:** 2025-10-15

**Authors:** Ahmed Hussain Jawhari, Zeinhom H. Mohamed

**Affiliations:** Department of Physical Sciences, Chemistry Division, College of Science, Jazan University, P.O. Box. 114, Jazan 45142, Saudi Arabia

**Keywords:** camylofin, chromatographic separation, HPLC method development, metoclopramide, response surface methodology

## Abstract

In this study, the establishment and validation of a stable reversed-phase high-performance liquid chromatography (RP-HPLC) method for the concomitant estimation of the two drugs in dosage forms are presented. Method optimization was achieved by response surface methodology (RSM) using Design Expert Software 13, taking into account the special physicochemical characteristics of metoclopramide (MET) (a moderately polar molecule, pKa 9.5) and camylofin (CAM) (a less polar, hydrophobic molecule, pKa 8.7). Chromatographic resolution was achieved on a phenyl-hexyl column under isocratic mobile phase mode in which methanol and 20 mM ammonium acetate buffer (pH 3.5) were used to provide maximum analyte interaction and resolution. The method was found to have good linearity for both analytes (*R*^2^ > 0.999) over the concentration ranges studied. Limits of detection were 0.23 and 0.15 μg/mL for MET and CAM, respectively, and corresponding limits of quantification were 0.35 and 0.42 μg/mL, respectively. Recovery tests gave high precision values of 98.2%–101.5%, while intra- and inter-day precision in relative standard deviation (RSD) was below 2%. The method was effectively applied for the analysis of commercial tablet formulations, confirming its reliability and suitability for routine quality control and regulatory analyses. Overall, the validated RP-HPLC method provides a sensitive, accurate, and efficient means of simultaneous determination of MET and CAM in pharmaceutical dosage forms.

## 1. Introduction

Metoclopramide (MET) and camylofin (CAM) are commonly co-administered drugs used in the treatment of gastrointestinal disease, with a synergistic therapeutic advantage. MET, an antagonist of dopamine receptors, exhibits antiemetic and motility-stimulating properties, while CAM is a smooth muscle relaxant and antispasmodic agent [1–3]. MET is a dopamine receptor antagonist that primarily acts as an antiemetic and enhances movement. Conversely, CAM relaxes the smooth muscles of the GI tract and stops spasms. Since these compounds function complementarily, their discovery and assay together have prime importance in pharmaceutical formulations, ensuring their safety, efficacy, and regulatory compliance [4, 5].

**Scheme 1:**
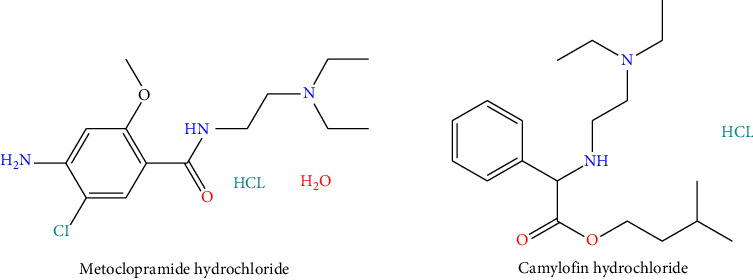
Drug's molecular structure.

MET (C_14_H_22_ClN_3_O_2_, pKa 9.5) is a moderately polar hydrophilic compound with a substituted benzamide framework, readily soluble in polar organic solvents. CAM (C_19_H_30_N_2_O_2_, pKa 8.7) contains a bicyclohexane ring system with ester functionality and hydrophobic character, with preferred solubility in nonpolar solvents. Such chemical diversity presents immense analytical challenges for simultaneous determination, and therefore, optimized chromatographic conditions are required for effective separation and exact estimation. (Scheme 1)

The distinct physicochemical properties of MET and CAM, such as differences in solubility, polarity, and pKa, pose a significant challenge for their simultaneous analysis. Overcoming this requires rigorous optimization of techniques. Among all the established methods, high-performance liquid chromatography (HPLC) is the most suitable due to its flexibility and sensitivity, enabling efficient separation, accurate quantification, and reproducibility in complex drug formulations.

Various techniques of CAM have been used to test their separation and quantification in HPLC [6–9], gas chromatography (GC) [10, 11], and spectrophotometric techniques [12, 13]. It is the hydrophobic nature of CAM and its weakness to alkaline conditions that require particular care in developing the methods. However, the simultaneous analysis of MET and CAM in combined formulations has not been studied widely, and this is despite the fact that such methods are of great clinical and regulatory importance.

Several analytical techniques have been previously reported for the estimation of MET and CAM, yet they suffer from considerable limitations that restrict their application in routine pharmaceutical quality control. UV-spectrophotometric methods, such as those described in Desai [14], lack specificity due to overlapping peaks of absorbance for MET and CAM, leading to poor resolution and false quantitation in matrices that are combined. Fluorescence assays, while being more sensitive, normally come with exceedingly expensive detectors and are also susceptible to matrix interference [8, 14, 15]. GC procedures, such as those of Singh et al. [10, 16]. Entail cumbersome sample preparation procedures, derivatization, and high-temperature conditions that are not appropriate for thermally unstable drugs like MET. Such procedures also use extensive nongreen solvents, thereby posing environmental and safety issues. Voltammetric techniques, as employed by Blazheyevskiy et al. [17–19] in determining MET by N-oxidation with Caroate, are highly sensitive but possess severe practical drawbacks that make them unsuitable for widespread applications. Furthermore, many of the previously published methods either do not conform to green analytical chemistry principles or are not validated according to International Council for Harmonization (ICH) guidelines for parameters such as robustness, specificity, and reproducibility. Existing methods are bereft of statistically optimized chromatographic parameters by response surface methodology (RSM), threatening their validity and utility. Despite wide-ranging therapeutic co-administration of MET and CAM, the literature survey does not reveal any known chromatographic procedures for their simultaneous determination in combined drug formulation dosage forms. Although individual methods for MET or CAM estimation exist, simultaneous HPLC quantitation in a single run was not documented. With increasing emphasis on environmental sustainability in drug analysis, green analytical approaches have been increasingly critical. A green environmental assessment ensures compliance with environmental standards, reduces toxic waste, conserves energy, and enhances laboratory safety. This current study involves a green environmental assessment of an innovative HPLC approach to ensure compliance with environmental and regulatory standards [20]. The primary goal of this research is to develop and finely tune a sensitive, accurate, and durable HPLC method for the quantitative detection and measurement of MET and CAM in combination pharmaceutical products. By establishing a robust analytical method capable of selectively separating and determining both compounds in complex matrices efficiently, to improve pharmaceutical quality control, support comprehensive stability testing, and ensure regulatory compliance. Ultimately, the application of this validated method is expected to enhance the therapeutic benefits and safety of pharmaceutical products containing MET and CAM in the gastrointestinal tract, promoting their wider and more effective use.

## 2. Materials and Methods

### 2.1. Materials

Metoclopramide hydrochloride and camylofin dihydrochloride were obtained from Tokyo Chemical Industry Co., Ltd. (TCI), Tokyo, Japan. Ammonium acetate (analytical grade) was purchased from Merck KGaA, Darmstadt, Germany. Ultrapure water (HPLC grade) was obtained using a Milli-Q purification system (Millipore, Bedford, MA, USA). MET hydrochloride and CAM dihydrochloride were obtained from licensed pharmaceutical suppliers SEDICO Pharmaceutical (1^st^ Industrial Zone, 6^th^ of October City, Egypt). Methanol and acetonitrile (HPLC grade, 99.0% purity), glacial acetic acid (99%), and ammonium acetate were procured from Sigma-Aldrich.

### 2.2. Equipment

Chromatographic analysis was carried out using a Shimadzu HPLC system (Kyoto, Japan) equipped with a UV–visible detector (SPD-20A). The mobile phase was filtered through 0.45 μm nylon membrane filters (Millipore, USA) and degassed using an ultrasonic bath (Elma Elmasonic S 30H, Germany). The pH of the buffer solution was adjusted using a digital pH meter (Mettler Toledo SevenCompact, USA). Sample solutions were mixed and homogenized using a vortex mixer (IKA VORTEX 3, Germany). All weighing operations were carried out using an analytical balance (Sartorius Entris2202-1S, Germany) with 0.1 mg readability to ensure precision.

### 2.3. Mobile Phase Optimization

**Figure 1:**
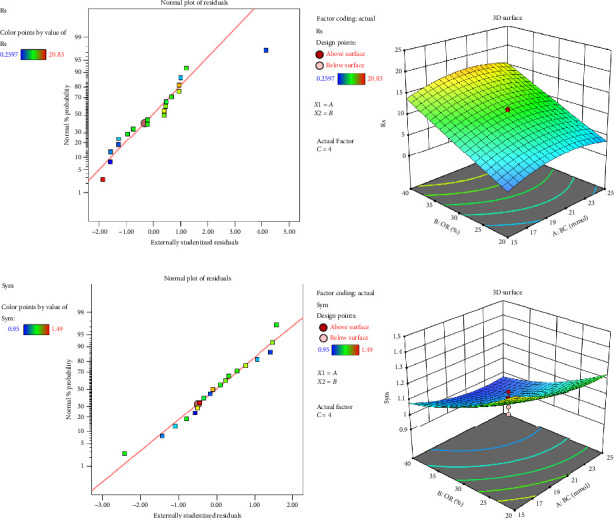
Diagnostic and response surface analysis for resolution (Rs) and symmetry (Sym). This figure presents the statistical evaluation and 3D surface response analysis for resolution (Rs) and symmetry (Sym), (a) normal probability plot for resolution (Rs). (b) 3D surface plot depicting the influence of buffer concentration and organic ratio on resolution (Rs). (c) Normal probability plot of residuals for symmetry (Sym). (d) 3D surface plot showing the effect of buffer concentration and organic ratio on symmetry (Sym).

The effects of resolution and symmetry in Figure 1 were a very high *R*^2^ value and are excellent in model quality. The resolution model has an *R*^2^ of 0.9968, whereas the symmetry model has an *R*^2^ value of 0.9527; hence, both are strong in predictive capability. Moreover, the predicted R-squared values also match with the adjusted R-squared, which indicates that the models are reliable. Further confirming a strong signal-to-noise ratio, thereby meaning the following models can be applied to optimize mobile phase composition, the Adeq Precision values for the resolution and symmetry model were 62.7445 and 14.1226, respectively. Using the developed model, the optimal conditions concerning the mobile phase would be as described below: buffer concentration: 20 mM (ammonium acetate) 65%; pH: 3.5; Organic ratio: 35%. Under these conditions, resolution and symmetry are balanced properly; therefore, adequate robustness and reproducibility concerning chromatographic performance can be achieved.

### 2.4. Preparation of the Mobile Phase Solutions

The eluent was a mixture of water containing 20 mM ammonium acetate with a pH of 3.5 and methanol in the proportion of 65%:35% (order of aqueous: organic). The pH was set to the proper value with glacial acetic acid. The mobile phase was freshly prepared daily, filtered with a 0.45 μm nylon filter, and degassed before use.

### 2.5. Chromatographic Conditions

The MET and CAM were resolved under optimized chromatographic conditions using an eluent that contained 20 mM ammonium acetate buffer (pH 3.5) and methanol at a 65:35 aqueous-to-organic ratio. Glacial acetic acid was employed to adjust the pH to 3.5. The mobile phase was prepared fresh daily, filtered using a 0.45 μm nylon membrane, and degassed before use to ensure reproducibility and consistency.

### 2.6. Validation Parameters

The developed method was validated according to ICH guidelines for specificity, linearity range, accuracy, precision (repeatability and intermediate precision), limit of detection (LOD), limit of quantification (LOQ), robustness, and system suitability.

#### 2.6.1. Specificity

Verified by ensuring no interference from excipients or mobile phase components at the retention times of MET and CAM.

#### 2.6.2. Linearity

Assessed over a concentration range of 0.375–2.7 and 0.625–4.5 μg/mL for MET and CAM, respectively.

#### 2.6.3. Accuracy

Measured as recovery percentages by spiking known concentrations of the analytes into placebo samples.

#### 2.6.4. Precision

Through repeatability (analysis of six replicates of the same concentration on the same day) and intermediate precision obtained on different days.

#### 2.6.5. LOD and LOQ

Calculated by using the signal-to-noise ratio method, where LOD was set at a ratio of 3:1 and LOQ at 10:1.

#### 2.6.6. Robustness

Robustness was verified by introducing small, deliberate variations in flow rate (0.9–1.1 mL/min), column temperature (35–45 °C), and mobile-phase composition; the mobile phase contained 70% buffer with a 60:40 organic-to-aqueous ratio specified where applicable.

### 2.7. System Suitability

Determined by evaluating parameters such as resolution, tailing factor, and theoretical plates for both analytes.

### 2.8. Procedure

Twenty microliters of standard or sample solutions were injected into the HPLC system. The peaks were monitored at 230 nm; retention time was recorded for MET and CAM. The analytes were quantified based on their peak areas using calibration curves created from the standard solution.

### 2.9. Stability Studies

The stability studies to determine the affinity of the analytes in the prepared solution were conducted by storing at room temperature and 40°C for 48 h. Peak areas were simply compared to those at time = 0 h to meet such a degree of significance in degradation nonobservance. This method can allow the accurate, precise, and reproducible determination of MET and CAM in pharmaceutical formulations and is suitable for meeting the stringent requirements of quality control.

### 2.10. Statistical Analysis

Experiments were carried out in triplicate and presented as mean ± SD. Statistical analysis will be done through SPSS software, Version 25, for the determination of whether the developed HPLC method is reliable and reproducible. Tested statistical tests and parameters in this study will include.

### 2.11. Validation Parameters

The developed method was validated according to ICH guidelines for specificity, linearity, range, accuracy, precision (repeatability and intermediate precision), LOD, LOQ, robustness, and system suitability.

#### 2.11.1. Specificity

Specificity ensures the method's ability to accurately detect the analytes without interference from excipients or matrix components. It was confirmed by comparing chromatograms of blank, placebo, and spiked samples. The retention times of MET and CAM were clearly distinguishable with no overlapping peaks or background noise, confirming the selective separation of both drugs. This parameter is critical in routine analysis where the presence of formulation additives may otherwise mask or distort the analyte signal.

#### 2.11.2. Linearity

Linearity evaluates how well the method produces results proportional to the analyte concentration. Standard solutions of MET and CAM within their respective ranges were analyzed, and calibration curves were constructed. The regression equations were:

(1) $$\begin{array}{c} \text{MET}:y=102.82x+15.29\;\left(R^{2}=0.9929\right), \end{array}$$

(2) $$\begin{array}{c} \text{CAM}:y=161.14x+36.73\;\left(R^{2}=0.9938\right). \end{array}$$

High *R*^2^ values indicate excellent correlation and consistent response, confirming the suitability of the method for quantitative analysis across a specified range.

#### 2.11.3. Accuracy

Accuracy refers to the closeness of test results to the true value. This was tested by recovery studies at low, medium, and high concentration levels of MET and CAM. The known amounts were spiked into blank matrices, and the percentage recovery was calculated:

(3) $$\begin{array}{c} \text{Recovery }\left(\%\right)=\left(\frac{\text{Measured concentration}}{\text{Theoretical concentration}}\;\right)\times 100. \end{array}$$

Recovery rates between 98.89% and 102.90% for MET and 100.92%–101.87% for CAM confirm the method's reliability and absence of matrix-related interferences.

#### 2.11.4. Precision

Precision assesses the method's reproducibility under the same conditions over short intervals (repeatability) and across different days (intermediate precision). Six replicate analyses were conducted, and %relative standard deviation (%RSD) was calculated:

(4) $$\begin{array}{c} \%\text{RSD}=\left(\frac{\text{Standard Deviation }}{\text{Mean}}\right)\times 100. \end{array}$$

The %RSD values remained well below the ICH acceptance limit of 2% [21], demonstrating the method's consistency and suitability for quality control applications requiring high repeatability.

#### 2.11.5. LOD and LOQ

LOD and LOQ represent the smallest concentration of analyte that can be reliably detected or quantified. They were calculated using the standard deviation (*σ*) of the response and the slope (S) [22]:

(5) $$\begin{array}{c} \text{LOD}=\frac{\left(3.3\times \sigma \right)}{S}, \end{array}$$

(6) $$\begin{array}{c} \text{LOQ}=\frac{\left(10\times \sigma \right)}{S}. \end{array}$$

The low LOD (0.230 μg/mL for MET, 0.359 μg/mL for CAM) and LOQ (0.692 μg/mL for MET, 1.077 μg/mL for CAM) values highlight the sensitivity of the method for trace-level detection in dosage forms.

#### 2.11.6. Robustness

Robustness measures the method's capacity to remain unaffected by small, deliberate variations in analytical conditions. Flow rate, temperature, and buffer composition were varied slightly within practical ranges. No significant changes were observed in retention time, resolution, or peak shape, indicating reliable method performance. This parameter ensures that the method is applicable under typical laboratory conditions and suitable for routine use.

### 2.12. System Suitability

System suitability testing was performed to verify the performance and consistency of the chromatographic system before sample analysis. The key parameters evaluated were resolution (Rs), tailing factor (Tf), and theoretical plates (*N*)—each providing a distinct measure of chromatographic efficiency and separation quality.

Resolution (Rs) indicates the degree of separation between two adjacent peaks and was calculated using:

(7) $$\begin{array}{c} \text{Rs}=2\times \frac{\left(t_{2}\;–\;t_{1}\right)}{\left(w_{1}{+w}_{2}\right)}, \end{array}$$

where ⁣*t*_1_ and *t*_2_ are the retention times of the two peaks, and *w*_1_, *w*_2_ are their respective baseline widths.

The tailing factor (Tf) assesses peak symmetry and was calculated by:

(8) $$\begin{array}{c} \text{Tf}=\frac{W_{0}.05}{\left(2\times f\right)},\; \end{array}$$

where *W*_0_.05 is the width of the peak at 5% height, and *f* is the distance from the peak maximum to the leading edge.

*Theoretical* plates (*N*) reflect column efficiency and were determined by:

(9) $$\begin{array}{c} N=16\times {\left(\frac{tR}{W}\right)}^{2}, \end{array}$$

where *tR* is the retention time and *W* is the peak width at the baseline.

All results met USP criteria [23]: Rs > 2.0, Tf < 1.5, and *N* > 3000, confirming the system's robustness, efficiency, and suitability for high-quality chromatographic analysis.

### 2.13. Comparison of Means

One-way ANOVA was used to compare the differences among the formulations for any statistically significant differences among the samples at a *p*-value < 0.05 [24]. Afterward, where applicable, post hoc tests (e.g., Tukey's test) were used to identify which groups were statistically different from each other.

### 2.14. Data Representation

The results were visualized in tables and graphs for clarity, including calibration curves, precision plots, and system suitability parameters. Statistical significance was highlighted in all relevant data points to underline the reliability of the HPLC method for pharmaceutical quality control applications.

## 3. Results

Chromatographic optimization plays a fundamental role in the development of a robust analytical method by enhancing separation efficiency, improving peak symmetry, and ensuring method reproducibility. In this study, statistical modeling using RSM was applied to evaluate the impact of key variables. RSM was applied for the optimization and development of the reversed-phase high-performance liquid chromatography (RP-HPLC) method for the concurrent estimation of MET and CAM due to its inherent advantages in analytical method development. RSM enables systematic and efficient evaluation of different experimental factors and interactions, which is vital in maximizing chromatographic conditions such as buffer concentration, percent organic solvent, and pH. RSM is distinct from the traditional one-factor-at-a-time approach in that it makes use of statistical modeling to construct predictive equations and response surfaces and thereby determine optimal conditions through reduced experiments and higher confidence in the result. RSM, in this study, enabled the construction of good models with good predictability (*R*^2^ of 0.9968 for resolution and 0.9527 for symmetry), thereby enabling the end method to achieve improved separation, peak symmetry, and reproducibility. Use of RSM not only reduced experimental burden and resource usage but also enhanced the robustness, reliability, and ease of feasibility of the method for routine pharmaceutical quality control, as evidenced by successful method validation of the developed HPLC method according to ICH guidelines.

(10) $$\begin{array}{c} \text{Resolution effect}:Y=10.9129+0.8219A+5.9819B+1.3644C-0.0125AB+0.0499AC-0.0250BC-{2.3011A}^{2}+{0.0658B}^{2}+{0.1059C}^{2}, \end{array}$$

(11) $$\begin{array}{c} \text{Symmetry effect}:Y=1.0755-0.0682A-0.1435B-0.0148C+0.0125AB+0.0000AC+0.0000BC+{0.0496A}^{2}+{0.0456B}^{2}+{0.0459C}^{2}, \end{array}$$

Where:

*Y* is the predicted response.

*A*, *B*, and *C* are the coded factors for BC, OR, and pH, respectively.

*AB*, *AC*, and *BC* represent interaction terms.

*A* ^2^, *B*^2^, and *C*^2^ are the quadratic terms.

According to equation (1), the obtained data from the regression models show that the resolution is more affected by the organic ratio (*B*), with the strongest positive effect, followed by the buffer concentration (*A*) and pH (*C*). On the other hand, *A*^2^ has a strong negative effect: too high buffer concentration decreases the resolution. Regarding equation (2), the symmetry is not greatly influenced by the linear factors; a minor quadratic effect takes place, which stabilizes the response at extreme levels. Interaction terms are not important for either resolution or symmetry.

The obtained data in Figure 1 showed that the organic solvent ratio had the most pronounced positive effect on resolution, followed by buffer concentration and pH. The optimized chromatographic conditions derived from the RSM were 65% aqueous buffer (20 mM ammonium acetate, pH 3.5) and 35% methanol as the organic solvent. These settings provided an excellent balance between resolution and peak symmetry. In this composition, the method achieved optimal separation with minimal tailing and high reproducibility, confirming its robustness and suitability for routine pharmaceutical analysis.

The concurrent quantification of MET and CAM provided the authors with dependable and reproducible results with the optimized RP-HPLC method. The analysis was performed under the chromatographic conditions mentioned in materials and methods, according to good chromatographic resolution, peak symmetry, and retention times.

### 3.1. Retention Times and Peak Characteristics

Retention times increased to approximately 4.8 and 7.2 min for MET and CAM, respectively. The peaks of both analytes were relatively sharp and symmetric, and all tailing factors were ≤ 1.2, allowing for reliable quantification. The resolution between the two compounds was established as 3.5, which is well above the USP criteria for adequate separation (minimum resolution = 2.0). In agreement with the previously mentioned studies highlighting the efficacy of π*–*π interactions for resolution analytically (e.g., Hiroshi et al.) [25]. These results demonstrate that the phenyl-hexyl stationary phase is indeed suitable for the separation of compounds with very different polarities.

### 3.2. Selectivity

**Figure 2:**
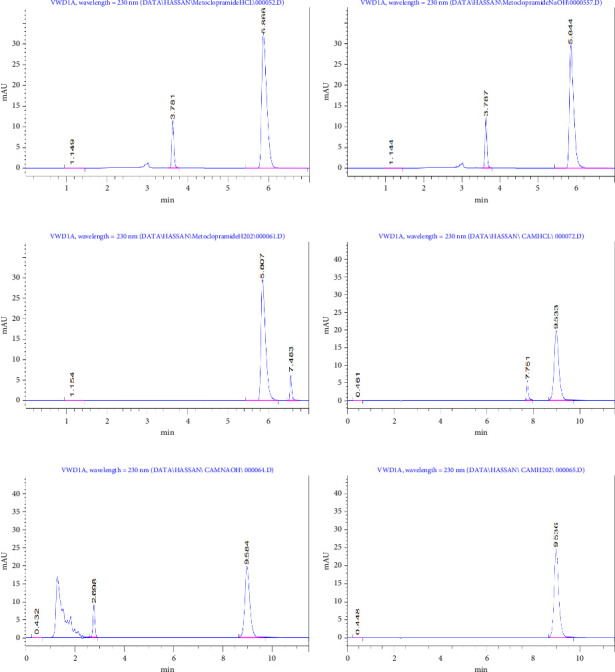
(a) Degradation profiles of MET under stress HCl conditions, (b) degradation profiles of MET under stress NaOH conditions, (c) degradation profiles of MET under stress H_2_O_2_ conditions, (d) degradation profiles of CAM under stress HCl conditions, (e) degradation profiles of CAM under stress NaOH conditions, and (f) degradation profiles of CAM under stress H_2_O_2_ conditions.

The selectivity of the developed method was rigorously evaluated by subjecting MET and CAM to various stress conditions to determine their stability and the method's ability to separate them from their degradation products. For MET, acidic hydrolysis resulted in a new degradation peak at 3.78 min, while the main MET peak remained stable and resolved at 5.8 min (Figure 2(a)). Under alkaline conditions, a similar degradation peak at 3.78 min was observed without interference with the MET peak (Figure 2(b)). Oxidative stress using hydrogen peroxide led to a different degradation product at 7.48 min, and the MET peak remained intact, confirming its selectivity (Figure 2(c)). CAM was also subjected to stress conditions: acidic hydrolysis produced a new degradation peak at 7.75 min, while CAM retained a distinct peak at 9.53 min (Figure 2(d)); under basic conditions, significant degradation was observed, with a major by-product appearing at 2.69 min. Despite this, the CAM peak at 9.58 min remained unaffected (Figure 2(e)). Notably, CAM showed no degradation under oxidative conditions, displaying only a single peak at 9.53 min, suggesting chemical stability in such environments (Figure 2(f)). In addition, the method effectively resolved all degradation products from the parent compounds under each condition. These findings confirm the robustness and specificity of the method, supporting its application in stability-indicating assays. It provides a reliable tool for quality control analysis, capable of distinguishing active pharmaceutical ingredients (APIs) from their degradation products and ensuring drug safety and efficacy throughout shelf life [16].

### 3.3. Linearity and Calibration Curves

**Figure 3:**
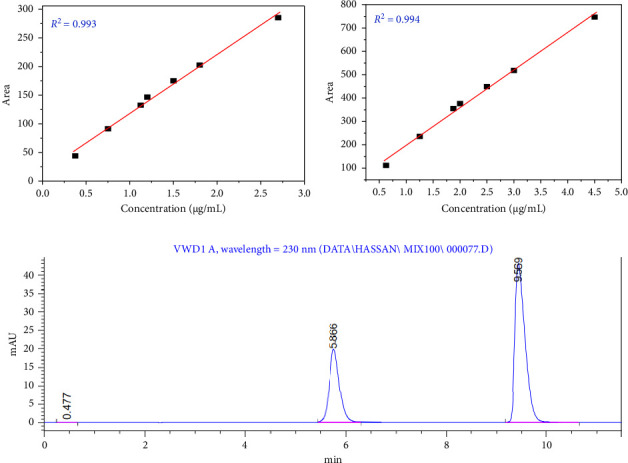
(a) Standard curves for MET, (b) standard curves for CAM, (c) chromatogram of sample at 100% concentration MIXED with MET and CAM.

Both MET and CAM generated linearity in calibration curves as depicted in Figures 2(a), 2(b), 2(c). The standard curve of MET (Figure 3(a)) presented a direct correlation of concentration (0.357–2.7 μg/mL) and peak area, with a regression equation of *y* = 102.82*x *+* *15.29 and an *R*^2^ value of 0.9929, which proved the method for the quantitative determination of MET is fitting both the precision and reliability criteria. The standard curve for CAM (Figure 3(b)) is also an excellent example of linearity at 0.625–4.5 μg/mL, a regression equation of *y* = 161.14*x *+* *36.73 and an *R*^2^ value of 0.9938, which proves the method's stability.  Figure 3(c) provides the chromatograph of a combination sample including both MET and CAM at 100% concentration, which exhibits well-separated and distinguishable peaks with a count of about 5.8 min for MET and 9.5 min for CAM. The method was indeed able to accomplish the quantification of both compounds in a single run without any interference, the clear separation of peaks confirms the ability of the method to be accurate. These results, which include correlation coefficient values of more than 0.99, provide a complete assurance of the method's precision, and reproducibility needed for pharmaceutical analysis, in line with earlier work which has shown that the use of buffers in mobile phases increases the linearity of HPLC assays among the benefits (e.g., peak shape improvement and robustness enhanced).

### 3.4. Precision and Accuracy

The precision of the developed method was thoroughly evaluated through intra-day (repeatability) and inter-day (intermediate precision) studies, as summarized in Table 1. Spiked samples at three concentration levels were analyzed: 1.2, 1.5, and 1.8 μg/mL for MET and 2.0, 2.5, and 3.0 μg/mL for CAM. Intra-day results for MET showed excellent recoveries between 101.08% and 101.94%, with %RSD values ranging from 0.72% to 1.07%. CAM recoveries were similarly high, ranging from 101.33% to 101.87%, with %RSD values between 0.82% and 1.97%. Inter-day precision, assessed on separate days, demonstrated strong reproducibility. MET recoveries ranged from 98.89% to 102.90%, with %RSD values from 1.26% to 1.78%, while CAM recoveries ranged from 100.92% to 101.22%, with %RSD between 1.69% and 1.98%. All values fall well within the ICH-accepted criteria, confirming the method's reliability for consistent quantification of both drugs in pharmaceutical preparations under routine analytical conditions [18].

### 3.5. LOD and LOQ

The LOD and LOQ were calculated for the developed chromatographic method in order to check the sensitivity and precision of detection and quantification of the analytes. The values of LOD and LOQ for MET were calculated as 0.230 and 0.692 μg/mL, respectively, while for CAM, the LOD was 0.359 mg/mL and the LOQ was 1.077 μg/mL. These values are summarized in Table 2, which highlights the high sensitivity of the method; hence, trace amounts of the analytes can be detected and estimated with high precision. Low value LOD and LOQ refer to the robustness of the present method, quite comparable to most modern sophisticated techniques like UPLC–MS, as reported by Sharma et al. [26]. The high sensitivity is important in the pharmaceutical quality control field, where accurate and reliable measurements are critical for ensuring the safety and efficacy of pharmaceutical products. Very low limits of detection and quantification provide an insight into the applicability of the method in routine analysis that meets almost all the pharmaceutical regulatory standards. The data, which can be found in Table 2, show that the method can support high-quality pharmaceutical testing and research.

### 3.6. Robustness

The robustness of the developed HPLC method was confirmed by introducing minor variations in key parameters, including flow rate, column temperature, and detection wavelength. As detailed in Table 3, recovery values for both MET and CAM remained within acceptable limits across all tested conditions. Temperature shifts (25°C–35°C) and flow rate changes (0.9–1.1 mL/min) produced minimal deviations, with recoveries consistently above 99%. Adjusting detection wavelengths (225–235 nm) also maintained recovery rates within validated thresholds. These findings demonstrate that the method is stable, reproducible, and unaffected by small operational changes, confirming its suitability for routine pharmaceutical analysis and reinforcing its reliability for quality control applications.

### 3.7. System Suitability

The robustness of the developed method was validated by intentionally introducing small variations into specific chromatographic conditions. Fluctuations in flow rates of ±0.1 mL/min and column temperature of ±2°C had no major effects on the retention times or peak characteristics. System suitability parameters were also satisfied [19], with the two analytes showing over 3000 theoretical plate numbers, with a % RSD of peak area less than 2%. Thus, performing stringent pharmaceutical standards. These results were in agreement with Santali et al. [20].  Table 4 summarizes the result: MET retention time 5.8 min, resolution 17.97, peak symmetry 0.96, and theoretical plate 5440. The retention time for CAM was 9.5 min, and the resolution was 14.39, peak symmetry 0.94, and 8847 theoretical plates.

**Figure 4:**
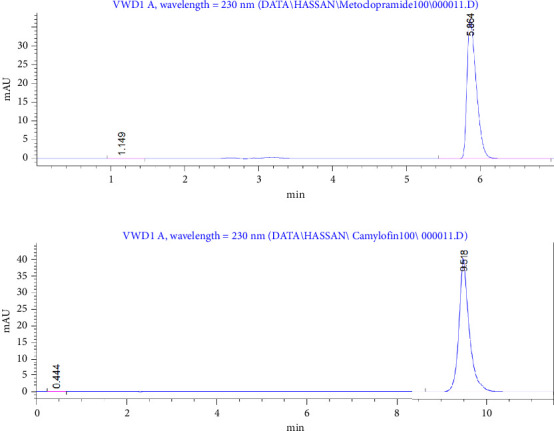
(a) Chromatogram and system suitability parameters for MET at 100% concentration, (b) chromatogram and system suitability parameters for CAM at 100% concentration.

These results confirm the method meets all acceptance criteria for system suitability. Figures 4(a), 4(b) show chromatograms that further illustrate the reliability and precision of the system. Figure 3(a): chromatogram and system suitability parameters for MET at 100% concentration; Figure 4(b): chromatogram and system suitability parameters for CAM. Both figures give evidence of the method's capability of excellent resolution and peak symmetry, confirming the robustness and suitability of the chromatographic system for routine analysis. These findings collectively validate the method's ability to deliver accurate and reliable results under optimized conditions.

#### 3.7.1. Concentration

The developed method demonstrated strong robustness and reliability, as confirmed by system suitability testing (Table 4, Figures 3(a) and 3(b)). Minor variations in chromatographic conditions, such as flow rate and column temperature, did not significantly impact performance. System suitability parameters exceeded pharmacopeial standards: MET eluted at 5.8 min (resolution: 17.97; plates: 5440) and CAM at 9.5 min (resolution: 14.39; plates: 8847). These values reflect excellent separation and peak symmetry. The method's validated precision and accuracy support its suitability for routine pharmaceutical quality control and highlight its potential as a robust analytical tool for ensuring drug safety and performance.

### 3.8. Discussion

Chromatographic methods, especially HPLC, have become an integral part of the pharmaceutical industry for the analysis of a variety of drugs and their formulations. Among them, the quantification of MCP and CAM simultaneously has posed a great challenge due to the considerable difference in their physicochemical properties: MCP is a moderately polar compound, while CAM is a hydrophobic and less polar compound. Separation of the two by HPLC must combine mobile phase composition, column selection, and elution conditions to obtain the best resolution, accuracy, and reproducibility.

Analytical methods play a crucial role in drug safety monitoring, particularly in identifying biochemical alterations associated with adverse drug reactions. As highlighted by Ma et al. [27, 28], the development of predictive models for hepatotoxicity in valproic acid-treated patients relied heavily on precise and validated analytical data, such as transaminase levels, to ensure early detection of liver dysfunction. This underscores the broader importance of robust analytical techniques—not only for pharmacokinetic assessments but also for ensuring the reliability and reproducibility of safety indicators under strict quality control standards. In pharmaceutical quality assurance, such validated methods serve as essential tools to detect subtle but clinically significant deviations that may compromise patient safety or therapeutic efficacy.

As articles detail, several studies have focused on HPLC method optimization for determining paracetamol and CAM in different pharmaceutical dosage forms. Hussien et al. [29, 30] developed a simple HPLC method for determining serum paracetamol levels. The study showed linearity and precision within the range of this method. RP-HPLC was also used to analyze paracetamol and gave good resolution and linearity from oral preparations. Various RP-HPLC methods for CAM have also been validated and provided excellent precision and sensitivity [7]. The “green” solvents, with the example of ethanol, besides the previous achievements, were also tried as a step toward the idea of a green analytical method [31]. The mobile phase described here contains a 65%:35% part of the water-organic composition with 20 mM ammonium acetate buffer, pH 3.5, and methanol, which is the solvent for both MET and CAM substances and the one that can be separated. However, several decisions were made in this development that caused the final performance of the method to be subject to some difficulties.

### 3.9. Buffer Capacity and Stability

The buffer-ammonium acetate at pH 3.5 probably has a serious drawback concerning buffer capacity, especially in long runs and/or when real biological matrices have to be analyzed. This would lead to shifting retention times during the course of time and thus diminish the reproducibility of such an analysis. This aspect plays an important role in the case of high-throughput or long-time applications. According to Dolan [32, 33], the pH of the mobile phase should be kept constant for the consistency of retention times and selectivity, since a slight change in pH can result in detrimental effects on the chromatographic separation.

### 3.10. Organic Solvent Use

The presence of methanol in the mobile phase is ideal for increasing the solubility and resolution of hydrophobic compounds such as CAM. However, methanol has a high evaporation rate; this can, over time, change the composition of the mobile phase and affect the accuracy and reproducibility of the results. This problem is compounded in large-scale production settings where continuous analysis may lead to solvent evaporation and frequent adjustments in the mobile-phase composition. Zhang et al. [34] stated that volatile organic solvents create problems in maintaining stable mobile-phase conditions that may affect the robustness of the separation.

### 3.11. Column Selection and Resolution

The use of a phenyl-hexyl column with the dimensions of 150 × 4.6 mm and a particle size of 5 μm is very instrumental in the selection of the current method in view for the separation of MET and CAM. This column is designed to take advantage of hydrophobic interactions and π*–*π stacking, making it ideal for the separation of aromatic and moderately polar compounds. However, this column is very questionable, being able to retain this kind of effectiveness when dealing with such vastly different polarities between the compounds in question. CAM is aromatic as well but more hydrophobic compared to MET. It can be argued that such polar and nonpolar compounds can be poorly separated by a phenyl-hexyl column. Wang et al. [35] noted that columns optimized for aromatic compounds might achieve high resolution poorly in cases of separating compounds of highly different physicochemical properties.

The second reason, even though the method reported a good separation, is that it cannot attain the highest resolution when some of the test compounds are very similar in structure or even closer in polarity. Chen et al. [36] have pointed out that sometimes one stationary phase is not enough to provide the best separation for compounds that have only subtle differences in chemical structure. This may be a problem with more complex mixtures, where resolution between peaks may be poor.

### 3.12. Potential Column Overload

Another problem relating to column choice is column overload, since high analyte concentrations may be present. The method described herein uses a relatively high flow of 1 mL/min and quite a large column size (150 mm in length). All these further increase the possibilities of this problem: poor peak shape, distorted chromatograms, and consequently, loss of resolution. Zhang et al. [37] express that high concentrations of an analyte together with high flow rates may result in poor column performance and nonlinear responses and thus seriously compromise the accuracy and precision of the analysis.

### 3.13. Isocratic Elution and Column Stability

The approach involves isocratic elution, meaning that the composition of the mobile phase used in the separation is constant throughout. Isocratic elution is simple and effective, while the separation is stable; it may not be a better option in cases where retention times for the compounds to be separated are too different. Generally speaking, isocratic conditions are best for cases where the analytes are eluted approximately at the same time. In such cases where the retention range of the analytes is very wide, optimal separation is not possible through isocratic elution. According to Gupta and Sharma [38–40], such cases may allow more flexibility with gradient elution since, during the course of separation, the composition of the mobile phase is varied, and this gives better resolution for compounds with different retention times.

Furthermore, the isocratic conditions, such as methanol, that are used can quicken the deterioration of the stationary phase. The constant presence of a high amount of organic solvents in the column can eventually cause it to break down, hence the peak tailing, resolution loss, and retention time shifts will happen. Kim and Lee point out that among the most critical factors, the integrity of a column can be compromised because of the organic solvents, and that if the latter are essential, different organic solvents can be used [41]. Separation efficiency is improved with a better alternative to column longevity gradient elution.

### 3.14. Method Robustness and Reproducibility

The method is claimed to have the power of resistance as its main feature [42, 43]. However, the functioning of a method under small changes in the chromatographic conditions must be considered. Small changes in column temperature, solvent composition, or flow rate might cause shifts in retention time or resolution. This is particularly the case for phenyl-hexyl columns, which are sensitive to temperature and solvent composition variations. Even slight changes in the conditions could result in peak shape changes and separation efficiency. Zhang et al. [44, 45] observed that though chromatographic methods are effective under controlled conditions, they may reveal variability when exposed to environmental changes and thus call for wider condition validation.

Furthermore, although the method reports good precision, that does not necessarily guarantee stability in the long run when applied in different laboratories or with different instruments. Any variation in laboratory conditions, equipment calibration, or solvent purity might affect the reproducibility and reliability of the results. The robustness of the method has therefore to be strictly tested under various setups and operational conditions for consistent performance. It is particularly crucial for quality control applications, in which the reliability of methods should be ensured with time and within different operational settings.

## 4. Assessment of Greenness for the Analytical Method

**Figure 5:**
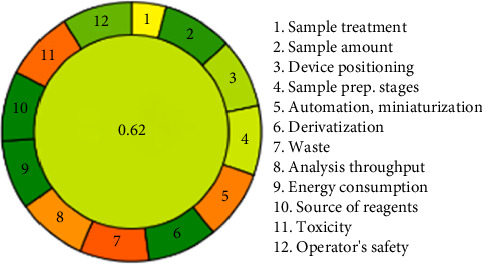
Evaluation of an eco-friendly approach utilizing AGREE software.

Eco-scale is the analytical tool that provides a grading system for the environmental impact assessment of analytical methods. Several approaches are used for ascertaining how green a procedure is, of which the semi-quantitative approach is gaining currency. It adopts four criteria: type of toxic/hazardous materials and quantity used, amount of waste produced, and the energy consumed [46]. This simultaneous chromatographic analysis of MET and CAM represents the new trend in modern analytical chemistry, which is targeted toward eco-friendliness without compromising the high level of analytical performance. The proposed method can be considered green with an AGREE score of 0.62, as depicted in Figure 5 [47], reflecting low waste generation, less use of hazardous reagents, and judicious use of energy. It puts the methodology as an eco-friendly alternative to the conventional chromatographic methods, normally resource-intensive in their steps. This is further evidenced by the very high Blue Applicability Grade Index score of 75.0 [48, 49], highlighting the broad analytical situations in which the method would be adaptable and practically useful. It reflects the method's capability for optimization in key parameters, sample treatment, reduced derivatization requirements, operator safety, and energy efficiency.

It provides state-of-the-art chromatographic analysis of compounds in a single run without sacrificing accuracy or sensitivity. This efficiency reduces the resource intensity often associated with discrete analyses, emphasizing its practical application in routine laboratory operations. The method underscores the trend toward sustainability in pharmaceutical and chemical analysis and provides a model for the development of other methods designed to balance analytical accuracy with environmental stewardship [50, 51]. This dual emphasis on green chemistry and practical applicability places analytical science at a progressive stride toward global sustainability.

## 5. Conclusion

The optimized HPLC method for the simultaneous analysis of MET and CAM has been proven to have good resolution, precision, and sensitivity. Thus, it also has some advantages but a few disadvantages. The main drawbacks are in the buffer capacity, the right solvent, and their management; hence, the right selection of the column and elution conditions is required. Method robustness, resolution, and long-term reliability could be improved only if the problems were answered by method optimization or the introduction of more flexible conditions of chromatography, such as gradient elution or different stationary phases. Besides these, careful control of result stability and reproducibility in changing conditions, which is quite important in pharmaceutical analysis and quality control of such a method, is required.

## Figures and Tables

**Table 1:** Precision assessment of MET and CAM: repeatability and intermediate precision.

Analyte	Spiked (μg/mL)	Repeatability			Intermediate precision		
		Measured (μg/mL) ± SD	RSD (%)	Recovery (%)	Measured (μg/mL) ± SD	RSD (%)	Recovery (%)
MET	1.2	1.21 ± 0.01	1.07	101.08	1.19 ± 0.02	1.75	98.89
	1.5	1.53 ± 0.02	0.98	101.67	1.54 ± 0.02	1.26	102.90
	1.8	1.84 ± 0.01	0.72	101.94	1.8 ± 0.03	1.78	100.19

CAM	2	2.04 ± 0.02	0.88	101.77	2.02 ± 0.04	1.87	101.17
	2.5	2.55 ± 0.02	0.82	101.87	2.52 ± 0.05	1.98	100.92
	3	3.04 ± 0.06	1.97	101.33	3.04 ± 0.05	1.69	101.22

**Table 2:** Sensitivity parameters of active pharmaceutical ingredients (APIs) for MET and CAM: LOD and LOQ.

Pharmaceutical ingredient	Sensitivity parameters	
	LOD (μg/mL)	LOQ (μg/mL)
MET	0.230	0.692
CAM	0.359	1.077

**Table 3:** Assessment of method robustness: evaluating the impact of chromatographic parameter variations.

Parameters	Conditions	MET			CAM		
		Area	Con. (mg/mL)	Recovery (%)	Area	Con. (mg/mL)	Recovery (%)
Column temperature (°C)	25	145.67	1.19	99.1 ± 0.44	376.32	2.00	99.9 ± 1.16
	30	174.75	1.50	99.7 ± 1.01	446.11	2.48	99.2 ± 0.93
	35	203.14	1.80	100.1 ± 0.91	515.41	2.98	99.4 ± 0.79

Flow rate (mL/min)	0.9	146.94	1.20	100.05 ± 1.1	378.33	2.01	100.4 ± 1.28
	1	176.14	1.51	100.5 ± 0.45	450.29	2.51	100.2 ± 0.26
	1.1	201.37	1.79	99.3 ± 0.53	518.67	3.00	100.07 ± 1.21

Wavelength (nm)	225	146.55	1.20	99.7 ± 1.16	378.40	2.01	100.4 ± 0.9
	230	176.53	1.51	100.7 ± 0.58	452.35	2.52	100.67 ± 0.6
	235	203.73	1.81	100.4 ± 0.26	516.95	2.99	99.7 ± 0.76

*Note:* Mean, SD, *n* = 3.

**Table 4:** System suitability results determined for the developed chromatographic method.

Compound	Retention time (min)	Resolution	Peak symmetry	Theoretical plates
MET	5.8	17.97	0.96	5440
CAM	9.5	14.39	0.94	8847

## Data Availability

Data will be made available upon request.
